# Depressive Symptom Profile Transitions From Midlife to Late Life in the Wisconsin Longitudinal Study

**DOI:** 10.1093/geroni/igaa057.1189

**Published:** 2020-12-16

**Authors:** Kyle Carr

**Affiliations:** Boston College, Chestnut Hill, Massachusetts, United States

## Abstract

Late-life depression is a significant public health problem among the growing elderly population in the United States. Structural social capital has previously been associated with lowering depressive symptoms in later life, but these results have been inconsistent. However, few studies have investigated this association when investigating different subtypes of depression. The current study used data from the Wisconsin Longitudinal Study (WLS) of 3,197 respondents to examine how structural social capital influences baseline depression statuses and transitions in these depression statuses. Latent class and latent transition analysis (LCA/LTA) were used to identify latent statuses at two time points in the WLS – 1992 and 2011 – as well as transitions between those statuses. Four depression statuses were identified at both time points: Very Depressed, Depressed and Lonely, Agitated and Restless, and Not Depressed. Gender, self-rated health, total assets, structural social capital measures, and polygenic score for depression were all predictors of baseline depression statuses. Transitions between depression statuses were associated with two forms of structural social capital – social support and social involvement. These findings add to the increasing number of studies investigating subtypes of depression in older adults as well as to scholars examining the association between structural social capital and depressive symptoms. Results suggest possible social and behavioral factors policymakers can use to identify risk of depression in mid-life and areas of intervention to improve depressive symptoms for aging adults.

